# Assessment of CoQ10 Dietary Intake in a Mediterranean Cohort of Familial Hypercholesterolemia Patients: A Pilot Study

**DOI:** 10.3390/nu17223512

**Published:** 2025-11-10

**Authors:** Teresa Sanclemente, Alicia Carazo, Tania Silvestre-Muñoz, Julio Montoya, Eduardo Ruiz-Pesini, José Puzo, David Pacheu-Grau

**Affiliations:** 1 Facultad de Ciencias de la Salud y del Deporte, Universidad de Zaragoza, 22002 Huesca, Spain; tsanclem@unizar.es (T.S.);; 2 Unidad de Lípidos, Servicio de Bioquímica Clínica, Hospital General Universitario San Jorge de Huesca, 22004 Huesca, Spainjpuzo@unizar.es (J.P.); 3 Departamento de Bioquímica, Biología Molecular y Celular, Universidad de Zaragoza, 50013 Zaragoza, Spain; jmontoya@unizar.es (J.M.); eduruiz@unizar.es (E.R.-P.); 4 Instituto de Investigación Sanitaria (IIS) de Aragón, 50009 Zaragoza, Spain; 5 Centro de Investigaciones Biomédicas en Red de Enfermedades Raras (CIBERER), 28029 Madrid, Spain; 6 Departamento de Medicina, Universidad de Zaragoza, 50009 Zaragoza, Spain; 7 Institute for Biocomputation and Physics of Complex Systems (BIFI), Universidad de Zaragoza, 50018 Zaragoza, Spain

**Keywords:** CoQ10, familial hypercholesterolemia, SAMS, statins

## Abstract

Background/Objectives: Coenzyme Q 10 is a lipid molecule that works as a mobile electron transporter in the mitochondrial respiratory chain and, in addition, plays the role of an antioxidant. Interestingly, CoQ10 synthesis in human cells derives from the mevalonate pathway, the same metabolic route that delivers endogenous cholesterol. Mutations leading to Familial Hypercholesterolemia (FH) alter the levels of CoQ10 production and remarkably, statin therapy associated muscular symptoms (SAMSs) might also be modulated by CoQ10 supplementation. CoQ10 is also provided by diet and only a few studies have calculated the dietary intake of this metabolite among populations. Methods: Here, we present our Spanish FH cohort (*n* = 261) and characterized relevant clinical, metabolic, and anthropometric parameters. Results: A cohort of 75.1% followed lipid-lowering treatment at inclusion, being the most prescribed drugs statin alone (32.7%) and statins combined with ezetimibe (56.6%). The average time on statin treatment was 3.7 years. Interestingly, 22% of cohort patients presented with SAMS. In addition, we performed an exhaustive literature review to define for the first time the CoQ10 content present in food typically found in Spain or other southern-European countries and classified them from very rich (over 50 mg/kg) to very poor (<1 mg/kg). With this information, we calculated the daily intake of CoQ10 from a small group (12) of selected FH patients using a validated food-frequency questionnaire (FFQ) and determined a daily intake 9.72 ± 2.64 mg/day, different to other described populations. Conclusions: we discussed the relevance of exogenous CoQ10 for FH development and potential SAMS. Interestingly, this information can be extrapolated to define the regular CoQ10 intake of the Spanish population, especially when following the MedDiet.

## 1. Introduction

Coenzyme Q10 (CoQ10) is a fat-soluble ubiquinone that transfers electrons within the electron transfer chain and therefore plays a critical role in mitochondrial energy metabolism [1]. Interestingly, CoQ10 also works as an antioxidant molecule in different physiological scenarios. For instance, this lipid molecule has been shown to successfully prevent oxidation of plasma circulating LDL particles [2] and, therefore, has been postulated to be relevant for the development of cardiovascular disease in Familial Hypercholesterolemia individuals (FH) [3]. Interestingly, heterozygous FH (HeFH) is the most common autosomal dominant genetic disorder affecting humankind, being the most frequent mutations located in the LDLR (low density lipoprotein receptor), APOB (apoliprotein B) or PCSK9 (proprotein convertase subtilisin/kexin 9) genes [3]. LDL binding to its receptor controls the endogenous production of cholesterol through the regulation of HMGCR enzyme. Therefore, mutations in LDLR that difficult or abolish receptor binding have been associated with increased intracellular and plasma cholesterol levels in FH patients [4]. Interestingly, the mevalonate pathway converts acetyl-CoA not only into cholesterol but also into other metabolically relevant precursors such as CoQ10. Recent studies using cellular models for FH have determined that deficient binding of LDL to its receptor in the plasma membrane results in decreased CoQ10 de novo synthesis, total CoQ10 levels, and reduced gene expression of enzymes involved in CoQ10 biogenesis [5]. Thus, CoQ10 levels seem to be connected to cholesterol biogenesis and uptake.

Statins are the choice therapy for HF and have been shown to decrease mortality and coronary artery disease by inhibiting HMGCR and, therefore, lowering circulating lipids [6,7,8]. However, an important number of patients treated with these drugs (7–19%) developed muscular symptoms derived from statin treatment (SAMSs) [9]. It has been observed that statin treatment decreased circulating CoQ10 independently of statin solution, intensity, or treatment time [10]. Due to the established connection between mevalonate pathway dysregulation and CoQ10 biosynthesis, these decreased CoQ10 levels derived from statin treatment have been proposed as the causative agent of SAMSs [11,12,13]. However, the cellular CoQ10 source is not limited to the mevalonate pathway since a significant proportion could be acquired through exogenous sources. Therefore, CoQ10 supplementation has been assayed as a complementary strategy for statin-treated HF patients to improve SAMS [3]. However, the effectiveness of these treatments is controversial, since the results of the randomized controlled trials (RCTs) testing CoQ10 doses from 100 to 600 mg/day are contradictory [14]. In fact, whereas some studies were able to find significant improvement of SAMSs upon supplementation with doses from 100 to 200 mg/day [15,16,17], others with doses from 120 to 600 mg/day were not able to identify positive effects derived from CoQ10 concomitant treatment [18,19,20]. Nevertheless, the two most recent meta-analyses about CoQ10 supplementation effects concluded that this strategy was not beneficial for patients with statin-associated muscle pain, nor did it lead to better adherence to statin therapy [21,22]. These contradictory results may be due to several factors. First, as our previous analysis proposed, genetic variation between individuals may explain SAMSs [3]. Another reported factor is CoQ10 bioavailability due to its low water-solubility [23,24], therefore, it is difficult to estimate the real amount of the compound that reaches target tissues when used to improve SAMSs [25]. Finally, in none of the cases has the contribution of naturally occurring CoQ10 in the foods that make up individuals’ typical diet been considered.

Major dietary sources of CoQ10 include meats, fish, nuts, vegetable oils (and foods fried in these oils). Much lower levels of CoQ10 can be found in most dairy products, vegetables, fruits, and cereals [26,27]. Once inside enterocytes, dietary CoQ10 is loaded into chylomicrons. Consequently, chylomicrons in the blood carry the CoQ10 to the liver, where it is primarily loaded into LDL and VLDL lipoprotein particles. A much smaller quantity of CoQ10 is packaged into high-density lipoprotein cholesterol. Moreover, the platelets and leucocytes in the blood contain CoQ10. The erythrocytes contain a quite small amount of CoQ10. Regarding plasma half-life calculations, studies have shown that the maximum concentration of ingested CoQ10 is reached after 6 h, while the half-life of absorbed CoQ10 is approximately 33 h. Despite the partial evidence that CoQ10 may exert beneficial effects on the aforementioned statin-treated patients, and the relatively high number of studies that have assessed the content of CoQ10 in food [26,27], only a few studies have estimated the average dietary intake of CoQ10 in specific populations [28,29,30,31,32,33,34]. Interestingly, not only the pharmacological treatment but also following an adequate diet, such as the Mediterranean Diet pattern (MedDiet), plays a critical role in the treatment of FH by reducing cholesterol levels [35].

Therefore, assessing the contribution of dietary CoQ10 to the overall CoQ10 status in FH patients may help clarify its potential involvement in SAMSs and other metabolic features. In this context, the present study had two main objectives: (i) to develop and validate a methodology to estimate dietary CoQ10 intake based on a comprehensive food composition review and a validated food-frequency questionnaire; and (ii) to apply this methodology in a Spanish FH cohort to provide the first estimates of dietary CoQ10 intake in a Mediterranean population and explore its relationship with adherence to the MedDiet.

## 2. Materials and Methods

### 2.1. Patients’ Cohort

The cohort focuses on the study of patients who have been diagnosed with Familial Hypercholesterolemia in the province of Huesca (Spain). The inclusion criteria of the population making up the cohort were as follows:

(a) Patients at least 4 years old who attended the Unity of Lipids of the University Hospital San Jorge of Huesca (Spain);
(b) Those who have obtained a score greater than 6 according to the Dutch Lipid Clinic Network diagnostic criteria for Familial Hypercholesterolemia (Table 1) [36,37].

The inclusion process began in 2022 and remains active to date. The study comprised a large FH registry (*n* = 261) used for descriptive cohort characterization and a pilot subsample (*n* = 12) in which dietary CoQ10 intake was estimated. All patients signed an informed consent form approved by the local ethics committee (nº 14/2022) to participate in the present study. After this, data were collected (review of medical record and interview with the patient) to complete the medical history (sociodemographic and clinical information). The information included in the database is as follows:

1. Identification data:
  - Medical record number;
  - Laboratory number.
2. Demographic:
  - Date of birth and age at the time of inclusion;
  - Gender;
  - Ethnicity.
3. Clinical:
  - LDL-C max;
  - Lipid-lowering treatment;
  - Time of treatment;
  - Statin-associated muscle symptoms (SAMSs);
  - Cardiovascular accident;
  - Age of first cardiovascular event;
  - The results of genetic studies, where available, were recorded (type of mutation and affected gene).

### 2.2. Biochemical Data, Body Composition Analysis, and Dietary Intake Assessment

Fasting blood samples from selected patients were collected to determinate routine biochemical parameters (glucose, urea, creatinine, total proteins, albumin, total bilirubin, alanine transaminase (ALT), aspartate transaminase (AST), alkaline phosphatase (ALP), g-glutamyl transferase (GGT), lactate dehydrogenase (LDH), and creatine kinase (CPK)), and lipid profiles (total cholesterol (TC), high-density lipoprotein cholesterol (HDL-C), triglycerides (TG) and low density lipoprotein cholesterol (LDL-C)) according to the manufacturer’s instructions, by spectrophotometric methods using an AU5800 clinical chemistry analyzer (Beckman Coulter). Ions (sodium, potassium, and chloride) were measured by indirect potentiometry in the same analyzer. LDL-C was calculated using the Friedewald equation, except in the presence of hypertriglyceridemia (>300 mg/dL) or low LDL-C concentrations (<70 mg/dL). In this case, direct LDL-C was measured.

In the same visit, body composition analysis and dietary intake assessment were performed. The anthropometric measurements were carried out by a trained health professional. The weight and height of participants were measured with bare feet and wearing light clothing. Waist circumference (WC) was measured at the thinnest part of the waist. Afterwards, percentage body fat mass (%FM) was determined using a Tanita^®^ body composition analyser (SC-330MA). Body Mass Index (BMI) and Waist-to-Height ratio (WHtR) were calculated according to the formulas: BMI = weight (kg)/height^2^ (m), and WHtR = WC (cm)/height (cm). The WHO thresholds widely accepted in Europe were used to interpret BMI and WC results [38] while a cut-off of 0.5 was used to interpret WHtR results [39,40].

Participants were interviewed to obtain information on their usual food intake over the previous year using a 136-item semiquantitative food-frequency questionnaire (FFQ) previously validated in FH patients [41]. For each food item, frequencies of specified serving sizes were registered in nine categories that ranged from “never or almost never” to “6 times/day”. From this frequency of consumption, the amount in milliliters or grams/day of each item was calculated by multiplying the frequency (times/day) by the grams or mL per serving.

The validated MEDI-LITE score was used to establish the adherence degree to the MedDiet. It consists of nine items referring to the daily consumption of fruit, vegetables, cereals, meat and meat products, dairy products, alcohol and olive oil, and the weekly consumption of legumes and fish/seafood. As the scores are assigned for each of the items, a higher score means greater adherence to the MedDiet [42]. According to different authors, a value of 12 means good adherence to this pattern [43,44].

### 2.3. Coenzyme Q10 Intake Estimation

Since worldwide food composition databases do not provide information on the content of CoQ10 in foods, an exhaustive review of the data published in the scientific literature was performed to identify the amount contributed by the 136 items included in the FFQ. To do this, we started from the two reviews published to date [26,27] whose results were completed with specific searches in the main databases (Pubmed, ScienceDirect, Web of Science) for those foods of interest.

When several results were found for a type of food, the average of these was used. Similarly, when an item included more than one food, the associated quantity was expressed as the average value of the contents of each of them and was completed with the range of values. Following the cut-off points proposed by Pravst et al., each of the items was assigned one of the following classes of food CoQ10 sources: A: Very rich (over 50 mg/kg); B: Rich (10–50 mg/kg); C: Modest (5–10 mg/kg); D: Poor (1–5 mg/kg); E: Very poor (<1 mg/kg) [26].

Using the serving sizes considered in the FFQ, the amounts of Q10 provided per serving were calculated. Taking this information into account, it was proposed to assign each item to one of the following classes: I = Very good source (over 3 mg/serving); II = Good source (1–3 mg/serving); III = Modest source (0.5–1 mg/serving); IV = Poor source (0.2–0.5 mg/serving); V = Very poor source (<0.2 mg/serving).

Once each food item frequency of intake was obtained, CoQ10 dietary intake was computed using Excel. Dietary CoQ10 intake was derived by summing CoQ10 contribution from those food items of the FFQ for which published CoQ10 contents were available.

### 2.4. Statistical Analysis

As this work represents a pilot study with a limited sample size (*n* = 12), no formal hypothesis testing was performed. Instead, we focused on detailed descriptive statistics to characterize variability within the cohort and to identify potential trends worth exploring in future research. Given the exploratory scope, inferential analyses would have lacked statistical power and could have led to misleading interpretations. Quantitative variables are expressed as mean and standard deviation, and qualitative variables are expressed as total number and percentage (unless differently stated in table legends). Xi-square and Pearson correlation coefficient were calculated using GraphPad Prism v.8.

## 3. Results

### 3.1. Characterization of the FH Cohort

In May of 2024, the cohort comprised 261 people from the province of Huesca (Spain) with a probable or definite diagnosis of FH according to the Dutch Lipid Clinic Network criteria, i.e., a score of 6 or greater. Gender distribution showed that 44.1% of patients are male, whereas 55.9% are female. Most of them were Caucasian (99.6%), and only one (0.4%) belonged to another ethnicity. The average age within the cohort is 52.6 years, and it is similar between males and females. Interestingly, whereas mean maximum LDL cholesterol serum levels before lipid-lowering treatment were 218.9 mg/dL (and similar between males and females), the highest maximum LDL level recorded was 415.1 mg/dL, evidencing the heterogenicity of biochemical and clinical symptoms between FH patients. Indeed, 8.0% of patients had suffered a cardiovascular accident, i.e., acute myocardial infarction (AMI), ST-elevation acute coronary syndrome (STEACS), non-ST-elevation acute coronary syndrome (NSTEACS), or other ischemic heart disease. The mean age of the first cardiovascular event was 50.6 years (±11.8 years) and was similar in both genders (Table 2).

Lipoprotein(a) is an established, genetically determined risk factor for atherosclerotic cardiovascular disease and is frequently elevated in individuals with familial hypercholesterolemia (FH). Given its role in residual cardiovascular risk, independent of LDL levels, we have measured Lpa in the study cohort (Table 3).

To avoid further cardiovascular events due to atherosclerosis, 196 patients (75.1% of cohort) followed lipid-lowering treatment at inclusion. The most frequently prescribed drugs were either statin alone (32.7%) and statins combined with ezetimibe (56.6%). A minor number of patients were treated with statins combined with fibrates (1.5%) or combined with fibrates and ezetimibe (2%). In a group of patients, lipid lowering therapy had to be adapted, and, therefore, 6.6% and 0.5% of patients were treated only with ezetimibe or only with fibrates, respectively. The average time on statin treatment was 3.7 years (±2.8 years) (Table 4).

The most frequently prescribed statins were Rosuvastatin (56.0%) and Atorvastatin (28.0%). Other types of statins, such as Simvastatin, Pitavastatin, Pravastatin, or Fluvastatin, were less commonly administered as therapy with 8%, 7%, 3%, and 3% of patients treated with them, respectively (Table 5).

The total number of patients experiencing statin-associated muscle symptoms (SAMS) was 40 (22.0% of the total cohort), with a higher prevalence in females (57.5%) compared to males (42.5%) (Table 6). Regarding the distribution by statin type, Atorvastatin (30.0%) and Rosuvastatin (27.5%) were the most commonly associated with SAMSs in this cohort. Other statins, such as Pitavastatin, accounted for 17.5%, Simvastatin for 10.0%, and Fluvastatin for 2.5%. No cases of SAMSs were reported with Pravastatin. Additionally, 12.5% of patients showed intolerance to multiple statins. These findings suggest that more potent statins, such as Atorvastatin and Rosuvastatin, are linked to a higher incidence of muscle symptoms. Both are lipophilic statins, which may increase their muscle penetration and risk of SAMS. In contrast, Pravastatin is less potent and more hydrophilic, which could explain its better tolerance and absence of reported muscle symptoms in this cohort. Interestingly, chi-square analysis showed statistically significant differences between patients treated with only statins (34 out of 64) and patients treated with statins plus ezetimibe (6 out of 111) (*p* < 0.0001).

### 3.2. Pilot Subsample and Comparability to the Full Cohort

We randomly selected 12 patients (4 men and 8 women) from our FH cohort to be included in our pilot study to test our designed methodology. The average age was 52.8 years, and all patients were Caucasian, similar to the whole cohort. In the same way, the average maximum LDL cholesterol before treatment was 221 mg/dL (similar to the cohort). The highest LDL maximum recorded was 285.2 mg/dL. Only one participant had an episode of cardiovascular accident in their medical history. In addition, one patient (9.1%) had reported muscle symptoms derived from statin treatment (SAMS) and had to switch to a different lipid-lowering therapy (Table 7). This is consistent with observational studies in which SAMSs are reported by 5–20% of patients receiving statin therapy, and they are the main reason for discontinuation and statin non-adherence [9,45].

Next, we collected the genetic study information from our cohort subsample. A total of 7 out of 12 patients (58.3%) had their DNA analyzed to identify mutations responsible for FH. Interestingly, 71.4% (41% of total) of patients with a genetic study had a known variant of one of the genes involved in FH, and for 28.6% (16.7% of total) of studied patients, no causative FH mutation was identified. In addition, mutations in LDLR were the most common ones identified (80% of patients with a known FH mutation). In the same line of evidence, analysis in the whole cohort had evidenced that 53.4% of studied patients carried a FH known causative mutation, and, among them, the most frequent gene affected was the LDL-R.

Interestingly, 11 out of 12 selected patients (91.7%) were under lipid-lowering treatment. The most used treatment was statin + ezetimibe (72.7% of treated patients), whereas the remaining 18.2% and 9.1% were treated with only statins or only ezetimibe, respectively. Finally, the average time on statin treatment was 5.0 years (Table 8 and Supplementary Table S1). Interestingly, 50% of statin-treated patients were using Rosuvastatin, whereas 30% and 20% were using Atorvastatin and Simvastatin, respectively (Table 9).

Considering all parameters analyzed, we may conclude that the selected subsample showed similar demographic, clinical, and genetic parameters to the complete cohort.

### 3.3. Biochemical and Anthropometric Data of the Pilot Subsample

Biochemical analysis in our subsample showed that the mean total cholesterol value was 194.5 mg/dL (±42.5 mg/dL) and interestingly was higher in women (210.8 ± 36.7) than in men (162.1 ± 37.2). The mean triglycerides (TG) levels were 91.0 mg/dL (±40.6 mg/dL), and the LDL-C levels were 102.9 mg/dL (±33.9 mg/dL) for LDL-C. Similarly to cholesterol levels, LDL-C levels were higher in females, whereas TG levels were higher in males. Nevertheless, LDL-C levels were reduced to half of the average maximal levels observed when patients were included in the study and, therefore, show a good adherence to lipid-lowering therapies (Table 10). The therapy time of each patient included in the pilot study cohort, when biochemical parameters were determined, is shown in Supplementary Table S1.

Anthropometric and body composition measurements in our pilot study sample determined that the mean BMI was 24.6 kg/m^2^, and 50% of the sample was overweight or obese according to the WHO criteria for BMI values. 42% of FH patients subsample presented cardiovascular disease risk according to the values of WC and WtHR. Concomitantly, the same number of patients showed %FM values classified as high fat or obesity (Table 11).

### 3.4. Dietary CoQ10 Intake and Food Sources

To gain insights into the dietary patterns of our selected FH patients, we aimed to explore their adherence degree to the MedDiet. Therefore, we subjected our 12 FH patients to the MEDI-LITE questionnaire, obtaining an average value of the MEDI-LITE score of 11.1 ± 1.8 (range from 6 to 13). We conclude that our subsample of FH presented a good adherence to MedDiet and therefore, if we are able to calculate the CoQ10 intake of these patients, we would have a first estimation of CoQ10 content in the MedDiet.

Following an exhaustive data search from the literature to elucidate the content of CoQ10 present in the items present in the FFQ, we could determine the levels for 51 items belonging to the food groups: dairy products, eggs, meat and meat products, fish and seafood, fruits and vegetables, and vegetable fats. It has been reported that legumes and cereal products present CoQ10 contents below the detection limit [26,27], and there is no literature on the possible CoQ10 content of processed foods. No available information exists regarding the convenience food included in the FFQ. Regarding the content per kg of food, vegetable oils, except sunflower oil, in addition to rabbit flesh and heart, present a very rich CoQ10 content (more than 50 mg/kg and termed “A”). The rest of the meats or meat-derived products (except boiled ham) and oily fish, with between 10 and 50 mg/kg CoQ10, can be classified as rich sources (termed “B”). Other items included in this range are certain foods of plant origin, such as dried fruits, nuts, sunflower oil, and some fruits, such as cherries. Boiled ham, seafood (oysters, clams, and mussels), and vegetables as cabbage, cauliflower, broccoli, chard, or spinach are classified as modest CoQ10 containing (5 to 10 mg/kg and termed “C”). Other foods, such as dairy products, lean fish, eggs, fruits, and vegetables, have relatively lower levels and have been classified as low (termed “D”) or very low (termed “E”) CoQ10-containing foods. However, to assess the possible impact of these food items on the CoQ10 intake of FH patients, we need to take the serving size into account. So, we also generated a classification based on their CoQ10 content per serving. The group of meats and meat-derived products, together with oily fish and some vegetable oils, is consolidated as very rich/rich sources of this compound (more than 3 or between 1 and 3 mg/serving and termed “I” and “II”, respectively). It is interesting to note that some vegetables with a modest CoQ10 content per kg (cabbage, cauliflower, broccoli, chard, and spinach) are included in class II when considering the content per serving. Similarly, other foods with poor contents of CoQ10 per kg, such as some fruits (grapes, peach, apricot, nectarine), are classified as modest CoQ10 sources (0.5–1 mg/serving and termed III) when the serving is considered. However, some of the analyzed items retain their CoQ10 content classification as poor or very poor (0.5 to 0.2 or below 0.2 mg/serving and termed “IV” and “V”, respectively) even considering the serving (Table 12).

Based on the data of dietary intake obtained by the FFQ, we calculated the amount of CoQ10 taken by each patient per day. The average CoQ10 intake of the 12 participants was 9.72 ± 2.64 mg/day (range 6.03 to 15.93 mg/day). This intake was 10.01 ± 4.20 mg/day (range 6.03 to 15.93 mg/day) for men and 9.58 ± 1.83 (range 6.76 to 12.96) for women. As it is shown in Supplementary Table S2, these figures are higher than those reported for other European countries such as Denmark (3 to 5 mg/day) [28], Finland (4.62 mg/day) [29], and Poland (5.5 mg/day) [31] or Asiatic ones such as China (3.92 to 4.99 mg/day) [33,34], and Japan (4.48 mg/day) [30], whereas a study developed in USA showed a higher CoQ10 mean intake (19.2 mg/day) [32]. Regarding the food sources, almost 80% of CoQ10 ingested by our subsample originated from “meat” (i.e., meat, poultry, and meat-derived products), and dietary fats, mainly olive oil. The contributions of other groups, such as vegetables, fruits, and fish and seafood, were much lower (8.5%, 6.1%, and 5.7%, respectively). These irregular contributions among food groups are also found in some of the aforementioned studies. In the case of Denmark, “meat”, with 64%, marks a clear difference with respect to other foods. Similarly to our results, the Finnish study reported “meat” as the main source of CoQ10 (55%), followed by dietary fats (18%), mainly rapeseed oil [29]. In the Japanese studio, “meat” again occupied the first position (44%), although, in this case, it was followed by fish and seafood (22%) [30]. In contrast, one of the Chinese studies reported a predominance of dietary fats (52%) as a source of CoQ10, whereas “meat” occupied the second position (37%) [34]. Finally, the American studio reported an equitable contribution between “meat”, dietary fat, and those named by the authors as “fried food” [32].

Finally, to test whether there was a relationship between CoQ10 intake and the degree of adherence to the MedDiet pattern, we analyzed the correlation of these two parameters, but no statistically significant correlation was found (R^2^ = 0.5415, *p*-value > 0.05, Spearman correlation).

## 4. Discussion

### 4.1. Summary of Main Findings

In this study, we present an interesting cohort of patients with FH that will contribute to a better understanding of this pathology. One of the objectives to be developed in further studies is to try to elucidate the role that the corporal levels of CoQ10 may have on SAMSs and, specifically, the importance of exogenous contributions of this antioxidant, such as those obtained from the regular diet. Consequently, the first step to establish this relationship was to develop a valid methodology that would allow estimating the intake of CoQ10 in our cohort. This study is the first to compile all foods consumed in the MedDiet pattern for which their CoQ10 content is known and to show results on CoQ10 intake in individuals in a southern European country.

The demographic, clinical, biochemical, and genetic parameters of the pilot study sample were similar to the whole cohort. Remarkably, anthropometric measurements showed that an important percentage of analyzed patients present a high risk of cardiovascular events, even being under pharmacological treatment and showing a good adherence to the MedDiet pattern. The main contributors to CoQ10 intake were meat, poultry, and dietary fats—particularly olive oil—reflecting the characteristic pattern of the Mediterranean diet. No significant correlation was found between CoQ10 intake and the degree of adherence to the MedDiet as measured by the MEDI-LITE score, suggesting that CoQ10 intake depends more on specific food choices than on overall dietary quality indexes.

### 4.2. Comparison with Other Populations and Methodological Limitations

Interestingly, the estimated intake value for our sample (9.72 ± 2.64 mg/day) is higher than those reported for other European and Asian countries, but lower than the reported USA population. Some methodological issues in the calculation of these values could explain these differences, such as sample characteristics and databases of CoQ10 food contents. Regarding CoQ10 content databases, earlier studies [28,29,30] were based on values obtained from their own analyses, while studies published after 2010 used CoQ10 values from published literature, mainly those included in the review by Pravst et al. [26]. Our database represents a significant update by incorporating food analysis results from the last 15 years. Regarding the differences between our studies and reports from other countries, they differ in methodological aspects, such as the sample size analyzed to determine CoQ10 intake (National Dietary surveys considering the whole population in Denmark [28], Finland [29], Poland [31], and Japan [30], large adult samples in the two Chinese studies [33,34] or a relatively small sample of men in the US study [32]), the number of food items considered (being the American and the Chinese studies the ones with higher items considered [28,29,31,32,33,34]). In addition, the US study assumed CoQ10 content of some items based on their similarities with other compounds, such as fat content, which allowed them to include a group called “fried foods” as a source of CoQ10 [32]. However, daily intake of CoQ10 largely depends on the qualitative composition of the diet, i.e., on the typical dietary pattern of each country. In the case of Northern Finland, consumption of reindeer meat (100 g serving provides 16 mg CoQ10) is enormous [29]. Danish and Finnish diets present a low contribution of fish due to their low consumption [28,29] versus the high contribution observed in the Japanese diet [30]. Relatively high contribution of vegetable fats in other diets could be explained by the popularity of CoQ10-rich rapeseed oil [29], soybean oil [34], or olive oil, as in our sample. Consequently, although the methodology used may be responsible for the accuracy of the estimates and, to a limited extent of the disparity of CoQ10 intake, dietary habits, with a greater or lesser presence of foods rich in CoQ10, are the ones that best explain the differences in the results between different countries.

Despite the novel contribution of this study regarding the dietary content of CoQ10, it is necessary to point out some limitations related to our dietary assessment method. Dietary data acquired by FFQs depend on participants recalling their eating habits in the last year, which relies on memory and is subject to recall bias that may limit accuracy. To minimize these limitations, the FFQ used in our study has been validated for the Spanish population and includes detailed portion size references [41]. Additionally, the FFQ was administered by trained nutritionists who showed food photographs to participants and could clarify doubts to ensure more accurate responses. Nevertheless, FFQs often overestimate the real intake and are subject to social desirability bias in dietary self-report [66,67]. However, it is expected that these errors will have relatively little effect on the results obtained on CoQ10 intake, given the profile of food groups that contribute to it. Indeed, the foods that contribute the most to our final figure are those that are most likely not to be overestimated, since there is ample evidence of their negative effect on LDL-C levels [68] and, therefore, are foods not recommended in the context of HF [35]. Consequently, we can venture that the values obtained are valid. Another limitation of this pilot study is the absence of formal inferential statistics. However, our primary objective was to generate preliminary descriptive data and identify potential associations between dietary CoQ10 intake and metabolic variables in FH patients. These exploratory findings provide a valuable foundation for designing larger studies with sufficient statistical power to confirm these trends.

### 4.3. Physiological Implications

Most of the body’s daily requirement for CoQ10 is produced within the body, but some CoQ10 is acquired from food. It is estimated that the daily requirement for CoQ10, from both endogenous biosynthesis and food sources, is about 500 mg; this estimate is based on a total body quantity of about 2 g of CoQ10 and based on an average turnover time of 4 days in tissue [69]. As people increase in age, the ability of the body to synthesize its own CoQ10 decreases. In addition to the normal aging process, CoQ10 levels have also been shown to be depleted in several disorders and associated with certain pharmacotherapies such as statins [70]. Consequently, the optimal dietary intake of CoQ10 in these scenarios is not known with certainty, and it is not possible to conclude whether the amount estimated in our sample is sufficient to meet the body’s needs. However, it doubles the content of CoQ10 in some over-the-counter vitamins and dietary supplements. In addition, it accounts for 10% of the therapeutic doses used in previously published studies, which conclude that supplementation with CoQ10 could provide benefits on statin-induced myopathy [16,17,71].

In any case, it is important to reflect on the relevance of CoQ10 bioavailability. In fact, the lack of full understanding of the processes determining its bioavailability is postulated as the main reason for the contradictory results from studies investigating the effects of CoQ10 supplementation on health. It is known that intestinal absorption of CoQ10 is highly variable among individuals and, apparently, is independent of the form of CoQ10 (ubiquinone/ubiquinol) dispensed [72]. However, some other topics must be considered. Due to its hydrophobicity, in the duodenum, CoQ10 molecules are subjected to micellization to be prepared for intestinal absorption. Consequently, it is expected that a better CoQ10 absorption would take place in the presence of oil or fat-containing meals [73]. In addition, the body's CoQ10 absorption at a certain time [74] is a consequence of the limited amount of carrier molecules to access the enterocytes. Several studies have focused on estimating the impact that CoQ10 from food can have on plasma levels. Thus, Weber et al. are the first to show that dietary CoQ10 can contribute significantly to serum concentrations. 9 healthy male volunteers took 30 mg of CoQ10 naturally present in one serving of pork heart, and serum CoQ10 concentrations increased by 35% [28]. In a more recent study that examines how dietary habits influence plasma concentrations of CoQ10 in 60 healthy Japanese participants aged 20 to 65, it was found that total CoQ10 plasma levels were significantly lower in the vegetarian and vegan group compared to the omnivore group. This highlights the significant contribution of dietary sources, particularly meat and fish, to plasma CoQ10 levels, indicating that dietary choices play a crucial role in determining CoQ10 concentrations [75]. In a similar study, the effect of a two-week diet without meat or poultry, which are rich in CoQ10 content, on serum levels of this metabolite in 22 young women aged 20–21 years was investigated. Upon restricting the intake of meat and poultry, the participants’ average daily intake of CoQ10 from meals decreased by 48% (from 2.1 ± 0.6 to 1.1 ± 0.5 mg/day). Simultaneously, the average serum total CoQ10 levels decreased by 16% after the two-week dietary intervention. These results suggest that meat and poultry are significant sources of CoQ10 in the diet [76]. Altogether, since most of the CoQ10 intake in our sample of study comes from meat and poultry, vegetable fat, and, to a lesser extent, fish, it is expected that the dietary habits of our pilot study sample will lead to an increase in serum/plasma levels of CoQ10.

### 4.4. Dietary Recommendations for CoQ10 and FH

By using the a priori-defined dietary index “MEDI-LITE score” [42], we observed that our sample presented a good adherence to the MedDiet pattern. Therefore, we can conclude that they had healthy dietary habits, which is indeed common among FH patients [77]. However, no statistically significant correlation was found between diet quality and CoQ10 intake. This is consistent with the results of a prospective study of 8367 participants from the Multiethnic Cohort Study (MEC) that showed that better diet quality, established by HEI 2010, AHEI 2010, aMED, and DASH, correlated with serum levels of some carotenoids and tocopherols, but no significant associations were found for retinol and CoQ10 [78]. These authors justify the lack of correlation to the fact that only supplementation seems to influence plasma concentrations of CoQ10. However, we believe that these questionnaires may describe a dietary pattern, but do not reflect the intake of specific foods that are rich in this antioxidant and/or penalize the consumption of those that constitute the main dietary source of CoQ10 (i.e., “meat”). In line with this statement, thanks to the compilation of information on the content of CoQ10 in foods and the classification of these items according to their contribution per serving of this antioxidant, this work contributes to facilitating the recommendation of foods that, while complying with the evidence of being beneficial for the control of hypercholesterolemia, provide relevant doses of CoQ10. Thus, within classes I, II, and III, we find healthy foods such as poultry and rabbit, oily fish, vegetables as chard or Cruciferae, vegetable fats, and nuts (Table 12). Maximizing CoQ10 intake through food has the benefit of cost-saving and may possibly have long-term health effects, such as preventing SAMSs in HF patients.

## 5. Conclusions

Interestingly, we have provided the first insights into CoQ10 intake in the Spanish population and CoQ10 content in the Mediterranean diet. Our work might therefore be useful for future studies evaluating the role of the diet in FH patients and its potential modulating effect on statin-derived muscle symptoms. Although this is a pilot study, the application of this methodology in larger cohorts (ours and others) will allow to evaluate the influence of CoQ10 intake as a modulator of SAMS frequency or severity in FH patients. Interestingly, there is a need to take the dietary CoQ10 intake into account to carry out studies on genetic variants that modify other relevant aspects of CoQ10 metabolism or SAMS incidence (mtDNA variants that modulate mitochondrial function or statin binding, genes involved in the endogenous synthesis of CoQ10, etc.). In future work, it will be critical to complement dietary intake estimates with direct blood CoQ10 determinations to evaluate their correlation with intake levels. Furthermore, applying this methodology in nutritional or supplementation intervention studies will provide deeper insights into the role of CoQ10 status in the management of FH and SAMS.

## Figures and Tables

**Table 1:** Dutch Lipid Clinic Network diagnostic criteria for Familial Hypercholesterolemia.

Family History	Points
First-degree relative with known premature (<55 years, men; <60 years, women) coronary heart disease (CHD) OR	1
First-degree relative with known LDL-C >95th percentile by age and gender for country	1
First-degree relative with tendon xanthoma and/or corneal arcus OR	2
Child(ren) <18 years with LDL-C >95th percentile by age and gender for country	2
**Clinical history**	
Subject has premature (<55 years, men; <60 years, women) CHD	2
Subject has premature (<55 years, men; <60 years, women) cerebral or peripheral vascular disease	1
**Physical examination**	
Tendon xanthoma	6
Corneal arcus in a person <45 years	4
**Biochemical results (LDL cholesterol)**	
>8.5 mmol/L (>325 mg/dL)	8
6.5–8.4 mmol/L (251–325 mg/dL)	5
5.0–6.4 mmol/L (191–250 mg/dL)	3
4.0–4.9 mmol/L (155–190 mg/dL)	1
**Molecular genetic testing (DNA analysis)**	
Causative mutation shown in the LDLR, APOB, or PCSK9 genes	8
**Diagnosis (based on the total number of points obtained)**	
Definite FH: >8 points Probable FH: 6–8 points Possible FH: 3–5 points Unlikely FH: 0–2 points	

**Table 2:** Demographic and clinical data of cohort participants (*n* = 261). Quantitative variables are expressed as mean and standard deviation, and qualitative variables are expressed as total number and percentage.

	Total 261 (100)	Male 115 (44.1)	Female 146 (55.9)
**Age (years)**	52.6 ± 16.0	53.0 ± 14.3	52.2 ± 17.3
**Ethnicity**			
Caucasian	260 (99.6)	115 (100)	145 (99.3)
Other	1 (0.4)	0 (0.0)	1 (0.7)
**LDL-c máx (mg/dL)**	218.9 ± 56.1	218.4 ± 61.7	219.2 ± 51.5
**Highest LDL-c máx (mg/dL)**	415.1	403.6	415.1
**Cardiovascular accident**			
Yes	21 (8.0)	13 (11.3)	7 (4.8)
No	240 (92.0)	102 (88.7)	139 (95.2)
**Age of first cardiovascular event (years) (age range)**	50.6 ± 11.8 (25–78)	49.8 ± 9.4 (33–72)	52.1 ± 16.1 (25–78)

**Table 3:** Lpa values in the study cohort (*n* = 122). Data is presented as median, 25th and 75th percentiles.

	Total 122 (100)	Male 59 (48.4)	Female 63 (51.6)
**Lpa (mg/dL)**	52.1 [14.6–110.3]	60.7 [7.7–108.0]	49.2 [20.4–111.0]

**Table 4:** Lipid-lowering treatment in cohort patients (*n* = 196). Data expressed as total number and percentage.

	Total 196 (100)	Male 93 (47.4)	Female 103 (52.6)
**Statin treatment**	182 (92.9)	85 (91.4)	97 (94.5)
Monotherapy with statins	64 (32.7)	25 (26.9)	39 (37.9)
Statins + ezetimibe	111 (56.6)	57 (61.3)	54 (52.4)
Statins + fibrates	3 (1.5)	2 (2.2)	1 (1.0)
Statins + ezetimibe + fibrates	4 (2.0)	1 (1.1)	3 (2.9)
**Other treatments**			
Monotherapy with ezetimibe	13 (6.6)	8 (8.6)	5 (4.9)
Monotherapy with fibrates	1 (0.5)	0 (0.0)	1 (1.1)
**Years on statin treatment**	3.7 ± 2.8	4.0 ± 2.9	3.4 ± 2.8

**Table 5:** Types of statins used to treat cohort participants (*n* = 182). Data are expressed as total number and percentage.

Type of Statin	Total 182 (100)	Male 85 (100)	Female 97 (100)
Rosuvastatin	102 (56.0)	49 (57.6)	53 (54.6)
Atorvastatin	51 (28.0)	21 (24.7)	30 (30.9)
Simvastatin	16 (8.8)	7 (8.2)	9 (9.3)
Pitavastatin	7 (3.8)	3 (3.5)	4 (4.1)
Pravastatin	3 (1.6)	2 (2.4)	1 (1.0)
Fluvastatin	3 (1.6)	1 (1.2)	2 (2.1)

**Table 6:** SAMSs in cohort participants stratified by statin type. Data are expressed as total number and percentage.

Type of Statin	Total 40 (100)	Male 17 (42.5)	Female 23 (57.5)
Rosuvastatin	11 (27.5)	3 (27.3)	8 (72.7)
Atorvastatin	12 (30.0)	5 (41.7)	7 (58.3)
Simvastatin	4 (10.0)	1 (25.0)	3 (75.0)
Pitavastatin	7 (17.5)	5 (71.4)	2 (28.6)
Pravastatin	0 (0.0)	-	-
Fluvastatin	1 (2.5)	1 (100.0)	0 (0.0)
Intolerance to multiple statins	5 (12.5)	2 (40.0)	3 (60.0)

**Table 7:** Demographic and clinical data of the subsample for the pilot study (*n* = 12). Quantitative variables are expressed as average and standard deviation, and qualitative variables are expressed as total number and percentage.

	Total 12 (100)	Male 4 (33.3)	Female 8 (66.7)
**Age (years)**	52.8 ± 11.5	52.3 ± 16.2	53.0 ± 9.7
**Ethnicity**			
Caucasian	12 (100)	4 (100)	8 (100)
**LDL-c máx (mg/dL)**	221.0 ± 30.3	219.2 ± 26.4	221.9 ± 33.8
**Highest LDL-c máx (mg/dL)**	285.2	247.0	285.2
**SAMS**	11 (100.0)	4 (100.0)	7 (100.0)
Yes	1 (9.1)	0 (0.0)	1 (14.3)
No	10 (90.9)	4 (100.0)	6 (85.7)
**Cardiovascular accident**			
Yes	1 (8.3)	1 (25.0)	0 (0)
No	11 (91.7)	4 (75.0)	8 (100)
**Age of first cardiovascular event (years)**	61	61	-

**Table 8:** LDL-C lowering treatment of subsample subjects (*n* = 11). Qualitative variables are expressed as total number and percentage, and quantitative variables are expressed as mean and standard deviation.

	Total 11 (100.0)	Male 4 (36.4)	Female 7 (63.6)
**Statin treatment**	10 (90.9)	4 (100.0)	6 (85.7)
Monotherapy with statins	2 (18.2)	0 (0.0)	2 (28.6)
Statins + ezetimibe	8 (72.7)	4 (100.0)	4 (57.1)
**Other treatments**			
Monotherapy with ezetimibe	1 (9.1)	0 (0.0)	1 (14.3)
**Years on statin treatment**	5.0 ± 2.9	4.0 ± 2.6	5.0 ± 3.3

**Table 9:** Types of statins used to treat subsample subjects (*n* = 10). Data are expressed as the total number and percentage.

Type of Statin	Total 10 (100)	Male 4 (40)	Female 97 (60)
Rosuvastatin	5 (50.0)	2 (50.0)	3 (50.0)
Atorvastatin	2 (20.0)	1 (25.0)	2 (33.3)
Simvastatin	3 (30.0)	1 (25.0)	1 (16.7)

**Table 10:** Biochemical parameters measured in subsample subjects (*n* = 12). Data are presented as mean and standard deviation.

	Total 12 (100)	Male 4 (33.3)	Female 8 (66.7)
**Glucose (mg/dL)**	97.3 ± 23.0	88.3 ± 12.2	101.9 ± 26.4
**Urea (mg/dL)**	31.8 ± 10.5	37.0 ± 5.8	29.1 ± 11.6
**Creatinine (mg/dL)**	0.82 ± 0.22	1.05 ± 0.07	0.70 ± 0.15
**Total proteins (g/dL)**	7.2 ± 0.5	111.5 ± 66.6	80.8 ± 18.3
**Albumin (g/dL)**	4.4 ± 0.3	4.7 ± 0.2	4.3 ± 0.2
**Total bilirubin (mg/dL)**	0.67 ± 0.35	0.92 ± 0.47	0.55 ± 0.21
**Sodium**	139.8 ± 1.2	140.3 ± 1.0	139.5 ± 1.3
**Potassium**	4.45 ± 0.38	4.55 ± 0.53	4.40 ± 0.32
**Chloride**	104.8 ± 1.6	103.5 ± 1.3	105.4 ± 1.4
**ALT (UI/L)**	25.2 ± 10.2	32.8 ± 12.7	21.4 ± 6.6
**AST (UI/L)**	29.3 ± 6.4	33.0 ± 5.4	27.5 ± 6.4
**ALP (UI/L)**	74.3 ± 18.7	71.8 ± 20.8	75.6 ± 18.8
**GGT (UI/L)**	38.7 ± 48.8	72.8 ± 79.1	21.6 ± 7.7
**LDH (UI/L)**	184.5 ± 33.9	188.0 ± 21.2	182.8 ± 40.1
**CPK (UI/L)**	116.8 ± 96.5	119.8 ± 35.7	115.4 ± 118.6
**Total cholesterol (mg/dL)**	194.5 ± 42.5	162.1 ± 37.2	210.8 ± 36.7
**HDL-C (mg/dL)**	76.7 ± 15.5	68.8 ± 3.9	80.6 ± 17.8
**LDL-C (mg/dL)**	102.9 ± 33.9	75.2 ± 16.8	116.7 ± 32.2
**Triglycerides (TG) (mg/dL)**	91.0 ± 40.6	111.5 ± 66.6	80.8 ± 18.3
**TC/HDL-C**	2.6 ± 0.6	2.3 ± 0.4	2.7 ± 0.7
**Non-HDL-C (mg/dL)**	117.9 ± 39.2	93.3 ± 33.7	130.1 ± 37.6

**Table 11:** Body composition and anthropometric data in FH patients (*n* = 12). Quantitative variables are expressed as average and standard deviation and qualitative variables are expressed as total number and percentage.

	Total 12 (100)	Male 4 (33.3)	Female 8 (66.7)
**Height (cm)**	165 ± 8	170 ± 7	162 ± 7
**Weight (kg)**	67.4 ± 15.7	75.9 ± 11.2	63.2 ± 16.5
**BMI (kg/m^2^)**	24.6 ± 4.2	26.3 ± 2.9	23.8 ± 4.7
Underweight	1 (8.3)	0 (0)	1 (12.5)
Normal	5 (41.7)	2 (50)	3 (37.5)
Overweight	4 (33.3)	1 (25)	3 (37.5)
Obese	2 (16.7)	1 (25)	1 (12.5)
**Waist circumference (cm)**	85.0 ± 13.4	93.5 ± 12.0	80.8 ± 12.6
Healthy	7 (58.3)	2 (50)	5 (62.5)
No weight gain	2 (16.7)	1 (25)	1 (12.5)
Weight reduction	3 (25)	1 (25)	2 (25)
**Waist-to-Height Ratio**	0.52 ± 0.07	0.55 ± 0.07	0.50 ± 0.07
Healthy	7 (58.3)	2 (50)	5 (62.5)
Central obesity	5 (41.7)	2 (50)	3 (37.5)
**% Fat Mass (BIA)**	27.6 ± 8.3	23.7 ± 4.5	29.5 ± 9.3
Low	3 (25)	0 (0)	3 (37.5)
Healthy	4 (33.3)	2 (50)	2 (25)
High	4 (33.3)	2 (50)	2 (25)
Obesity	1 (8.3)	0 (0)	1 (125)
**% Muscular Mass (BIA)**	65.7 ± 12.4	63.3 ± 19.1	66.9 ± 8.8

**Table 12:** CoQ10 contents of FFQ items from literature search. * When more than one value is found, data is expressed as an average (range).

Foods	mg CoQ10/kg *	Class by mg/kg	Serving Size (g)	mg CoQ10 per Serving	Class by mg/Serving	References
**Dairy Products**						
Whole milk (UHT 3.5% fat)	1.70	D	200	0.340	IV	[46]
Semi-skimmed milk (UHT 1.6% fat)	1.16	D	200	0.232	IV	[46]
Skimmed milk (UHT 0.5% fat)	0.46	E	200	0.092	V	[46]
Milk cream (35% fat)	0.92	E	100	0.092	V	[46]
Yogurt (3.2% fat, natural/with fruits)	0.93 (0.72–1.13)	E	125	0.116	V	[46]
Yogurt (0.0% fat)	0.08	E	125	0.010	V	[46]
Cottage cheese or curd	0.62	E	100	0.062	V	[46]
Cream cheese	0.29	E	25	0.007	V	[28]
Cured or semi-cured cheese: Edam, Manchego, Emmental, Provolone, …	1.37 (1.20–1.58)	D	50	0.068	V	[29,30,47]
**Eggs**						
Eggs	1.43 (0.73–2.30)	D	60	0.086	V	[28,29,30]
**Meat**						
Chicken/turkey with/without skin	16.86 (11.44–25.00)	B	150	2.529	II	[28,29,30,48,49]
Beef	34.68 (23.47–48.78)	B	150	5.202	I	[28,29,30,49,50,51,52]
Pork	23.07 (13.08–45.00)	B	150	3.461	I	[28,29,30,48,49,52,53]
Lamb	24.80 (18.70–30.50)	B	150	3.720	I	[50]
Rabbit	103.00 (95.20–110.80)	A	150	15.450	I	[54]
Liver (chicken, beef, pork)	48.02 (21.63–116.2)	B	100	4.802	I	[29,30,48,49,50,51,53,55]
Other offal: heart (chicken, beef, pork)	130.63 (60.50–192.00)	A	100	13.063	I	[29,30,49,50,51,53]
Dry-cured ham	10.10	B	30	0.303	IV	[56]
Boiled ham	7.40	C	30	0.222	IV	[28]
**Fish**						
Lean fish: Flatfish, hake, cod, brill, red mullet, …	2.56 (1.80–3.70)	D	150	0.384	IV	[30,57]
Oily fish: Rainbow trout, mackerel, tuna, salmon, herring, sardine, …	10.73 (3.64–30.20)	B	150	1.394	II	[28,29,30,48,49,57,58]
Oysters, clams, mussels, …	5.75 (3.42–9.52)	C	60	0.345	IV	[30,57]
Octopus, squid, cuttlefish, …	4.00 (0.37–8.24)	D	200	0.801	III	[30,49,57]
Shrimps, prawns, crayfish, …	1.66	D	200	0.332	IV	[30]
Fish and shellfish, canned in oil (sardines, anchovies, bonito, tuna)	15.4 (14.9–15.9)	B	50	0.770	III	[29,30]
**Vegetables**						
Chard, spinach	10.00 (6.99–13.00)	C	200	1.999	II	[48,55]
Cabbage, cauliflower, broccoli	5.45 (2.70–10.93)	C	200	1.089	II	[28,29,30,48,49]
Tomato	1.65 (0.90–2.41)	D	150	0.248	IV	[29,48]
Carrot, pumpkin	2.97 (1.70–4.23)	D	200	0.297	IV	[29,48]
Beans	1.80	D	200	0.360	IV	[29]
Eggplants, zucchini, cucumbers	1.10 (0.08–2.20)	D	200	0.219	IV	[30,49]
Asparagus	2.16	D	200	0.432	IV	[30]
Onion	0.87 (0.67–1.07)	E	50	0.044	V	[30]
Garlic	3.45	D	2	0.007	V	[30]
French fries	0.69 (0.5–1.05)	E	150	0.104	V	[28,29,30]
Cooked and roast potatoes	0.69 (0.5–1.05)	E	200	0.138	V	[28,29,30]
**Fruits and Nuts**						
Orange, grapefruit, or clementine	1.74 (0.90–3.60)	D	150	0.261	IV	[28,29,30,48]
Banana	0,82	E	100	0.082	V	[30]
Apple, pear	1.20 (1.10–1.30)	D	150	0.181	V	[28,29,30]
Strawberries	0.96 (0.51–1.40)	D	60	0.057	V	[29,30]
Cherries, cherry blossoms, plums	13.34	B	150	2.000	II	[48]
Peach, apricot, nectarine	4.34	D	150	0.651	III	[48]
Kiwi	2.35	D	100	0.235		[48]
Grapes	4.56 (1.48–9.02)	D	150	0.683	III	[59]
Dates, dried figs, raisins, prunes	21.06 (8.32–67.43)	B	50	1.053	II	[60]
Almonds, peanuts, hazelnuts, pistachios, pine nuts	17.40 (4.99–26.19)	B	30	0.522	III	[30,48,55,61]
**Vegetable Oils**						
Olive oil	54.00	A	10	0.540	III	[62]
Virgin olive oil	76.58 (52.00–114.10)	A	10	0.766	III	[62,63,64]
Corn oil	139.10	A	10	1.391	II	[63]
Sunflower oil	12.20 (8.70–15.70)	B	10	0.122	V	[65]
Soy oil	188.45 (97.60–279.30)	A	10	1.885	II	[63,65]

Class by CoQ10 content per kg: A: Very rich (over 50 mg/kg); B: Rich (10–50 mg/kg); C: Modest (5–10 mg/kg); D: Poor (1–5 mg/kg); E: Very poor (<1 mg/kg) [26].

## Data Availability

The original contributions presented in this study are included in the article/Supplementary Material. Further inquiries can be directed to the corresponding author.

## Supplementary Materials

The following supporting information can be downloaded at: https://www.mdpi.com/article/10.3390/nu17223512/s1, Table S1. Lipid lowering treatment and years on treatment of each patient of the pilot subsample. Table S2. Comparison of total dietary intake of CoQ10 and the contributions of different food groups between countries. Expressed as mg/day (% of total intake).
