# Supplementary material for: Assessment of CoQ10 Dietary Intake in a Mediterranean Cohort of Familial Hypercholesterolemia Patients: A Pilot Study
Source: Nutrients. 2025 Nov 10;17(22):3512. doi: 10.3390/nu17223512 (PMC12655468; doi:10.3390/nu17223512)
Supplement: Supplementary file 1 [file nutrients-17-03512-s001.zip › nutrients-3955582-supplementary.pdf]

Patient	Treatment	Years on Treatment
1	Ezetimibe	9.1
2	Rosuvastatin	7.8
3	Rosuvastatin + ezetimibe	2.5
4	Simvastatin + ezetimibe	2.1
5	Simvastatin + ezetimibe	2.6
6	Atorvastatin + ezetimibe	8.3
7	Atorvastatin	2.2
8	Rosuvastatin + ezetimibe	3.2
9	Rosuvastatin + ezetimibe	4.1
10	-	-
11	Rosuvastatin + ezetimibe	5.9
12	Atorvastatin + ezetimibe	11.0

Supplementary Table S1. Lipid lowering treatment and years on treatment of each patient of the pilot subsample.

Country	COQ10 intake (mg/day)	Dairy products	Eggs	Meat	Fish	Vegetables	Fruit	Dietary fat	Other groups
**Spain**	9.72	0.02 (2.2%)	0.04 (0.4%)	4.99 (50.0 %)	0.54 (5.7 %)	0.85 (8.5%)	0.61 (6.1%)	2.50 (27.0%)	
**Denmark [28]**	3-5	0.1-0.5 (10.0%)	0.06-0.08 (1.6%)	2.2-3.2 (64%)	0.4 (8%)	0.3 (6%)	0.2 (4%)	0.2-0.3 (6%)	Cereals: 0-0.03 (<0.6%)
**Finland [29]**	4.62	0.36 (7.8%)	0.03 (0.5%)	2.55 (55.2 %)	0.4 (8.7 %)	0.26 (5.5%)	0.21(4 .4%)	0.83 (17.9%)	
**Japan [30]**	4.48	0.04 (0.9%)	0.03 (0.6%)	1.97 (44%)	0.99 (22%)	0.53 (12%)	0.26 (6%)	0.447 (10%)	Pulses: 0.224 (5%)
**USA [32]**	19.2	0.38 (2%)		5.18 (27%)	0.58 (3%)	0.58 (3%)	2.88 (15%)	4.80 (25%)	Fried food: 4.22 (22%) Cereals: 0.58 (3%)
**China [34]**	4.99	0.02 (0.4%)	0.06 (1.2%)	1.86 (37.3 %)	0.03 (0.6 %)	0.42 (8.4%)	0.04 (0.8%)	2.58 (51.7%)	
**Poland [31]**	5.5								
**China [33]**	3.92								

Supplementary Table S2. Comparison of total dietary intake of CoQ10 and the contributions of different food groups between countries. Expressed as mg/day (% of total intake).
